# Tacrolimus Induced Leukoencephalopathy and Stroke-Like Symptoms: Case Report

**DOI:** 10.1177/2329048X231171011

**Published:** 2023-04-18

**Authors:** Kelly M. Dopke, Nader El Seblani, Katherine Mercer, Sunil Naik, Gayatra Mainali, Dustin Paul

**Affiliations:** 1 12311 Penn State Health, Pediatric Neurology, Pediatric Neuromuscular Medicine, 30 Hope Drive, Suite 1300, Hershey, PA 17033, USA

**Keywords:** tacrolimus, tacrolimus-induced leukoencephalopathy, toxic leukoencephalopathy, pediatric neurology, magnetic resonance imaging

## Abstract

A 17-year-old female with sickle cell disease status post a recent stem cell transplant and on tacrolimus developed an acute expressive aphasia, dysphagia, and drooling. Brain MRI revealed diffuse restricted diffusion involving the bilateral corona radiata and areas of white matter in the right cerebral hemisphere most consistent with toxic leukoencephalopathy. Tacrolimus serum concentration was high at 19.3 ng/ml (ref 9-12 ng/ml) for which tacrolimus was discontinued. She was neurologically back at baseline 2 days later with the tacrolimus level improving to 8.2 ng/mL. Following discontinuation and the declining trend of her tacrolimus levels the patient returned to her neurologic baseline and was subsequently switched to mycophenolate mofetil for GVHD immunosuppression.

## Introduction

Toxic leukoencephalopathy (TL) was first described in 2001 as an encephalopathy affecting white matter due to an inciting causative agent such as therapeutic agents, radiation, illicit drug use, or occupational exposure to toxins.^1^ The clinical presentations of TL vary from mild cognitive impairment, psychiatric illness, to severe neurological deficits. Although the pathophysiology is unknown, it is proposed that white matter in the brain is vulnerable to toxic injury due to heavily myelinated fibers, limited blood flow, and high metabolic demands of glial cells, all of which can be targets of therapeutic drugs.^1,2^ Brain MRI has been crucial in identifying this syndrome at early stages. Although there are case reports about therapeutic agents causing TL, the literature indicates that tacrolimus induced TL has not previously been reported. Thus, we report a 17-year-old female with sickle cell anemia status post stem cell transplant on prophylactic treatment of tacrolimus for graft versus host disease (GVHD) who presented with stroke-like symptoms from tacrolimus induced TL.

## Case

A 17-year-old female with sickle cell anemia (hemoglobin SS) status post haploidentical bone marrow transplant from her biological mother 48 days prior and on tacrolimus for GVHD prophylaxis presented with acute onset expressive aphasia with associated drooling and dysphagia upon waking up from a 4-hour nap. After she arrived at our medical center, a stroke alert was activated. She scored 4 on the NIH stroke scale for her severe expressive aphasia and dysarthria. The mother denied any recent history of infection, chills, fever, loss of consciousness, or head trauma.

Examination revealed an awake and alert female with severe slurred speech and severe expressive aphasia who was able to respond well to all verbal commands, write, and read. Her cranial nerve, motor, and sensory examinations were all intact. The rest of her general exam was normal. Her vitals were stable, and the anthropometric parameters were appropriate for age.

**Figure 1.**
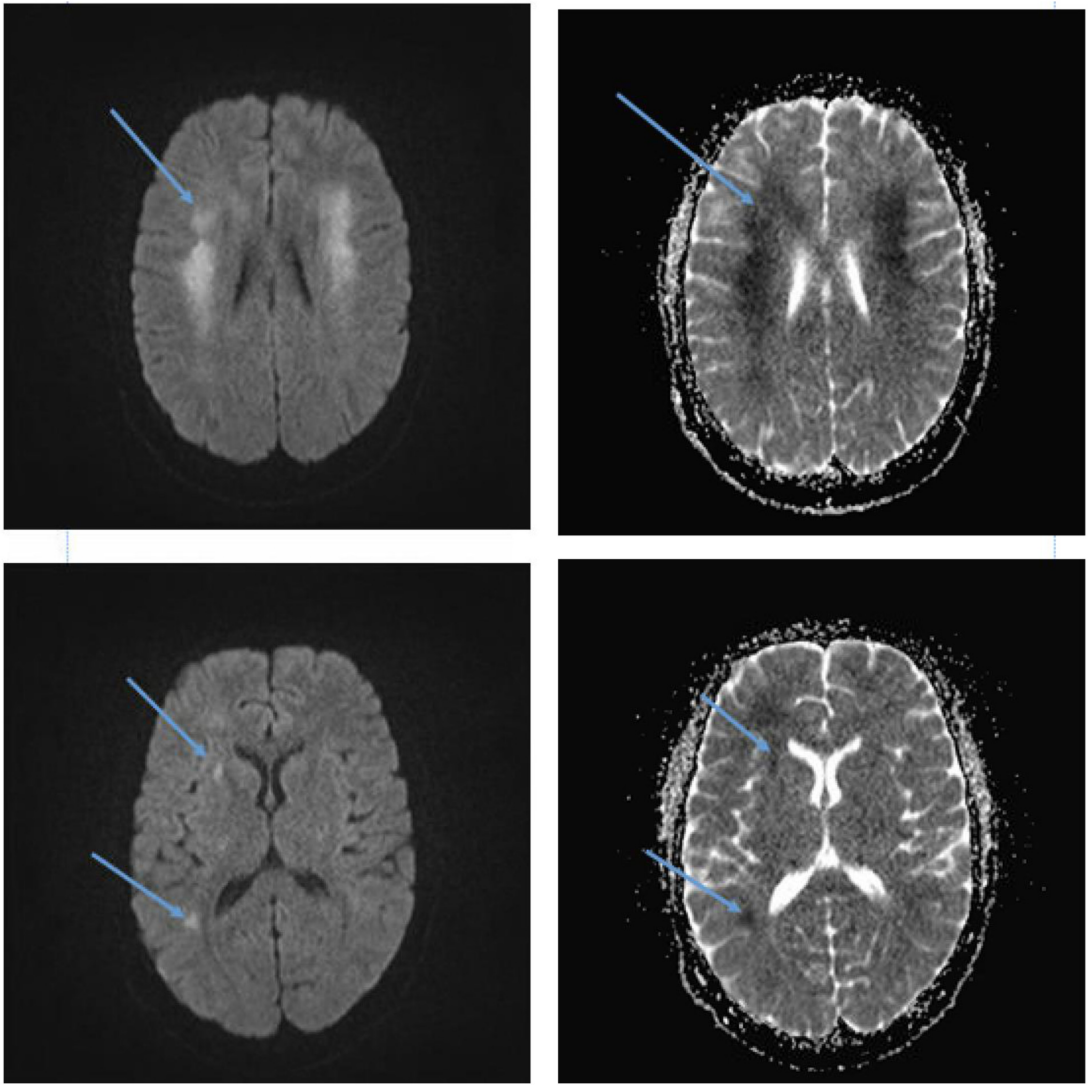
MRI brain DWI-ADC showing restricted diffusion involving the bilateral corona radiata (top images) and small areas of restricted diffusion involving the right anterior limb of the internal capsule and the right occipital white matter (bottom images).

Initial non-contrast head computed tomography (CT) scan, CT angiography, and CT perfusion were negative for acute intracranial pathology, bleeding, large vessel occlusion or flow-limiting stenosis. Thrombolytics were considered, yet the patient was not a candidate given that her last known well was >4.5hr prior (before she went to sleep) and thus outside the therapeutic window. Additionally, the use of thrombolytics remains controversial in those with sickle cell disease. Initial lab results for complete blood count, arterial blood gas, and blood chemistries were remarkable for a mildly elevated potassium level (5.5 mmol/L), decreased sodium level (133 mmol/L), and elevated liver function tests (alkaline phosphatase 344 unit/L, AST 75 units/L, and ALT 34 units/L). A lumbar puncture was also performed and returned normal with CSF findings showing a clear, colorless fluid with nucleated cell count 6/µL, RBC count 2/µL, glucose 42 mg/dL, protein slightly elevated at 47 mg/dL, myelin basic protein <2.0, and IgG index of 0.61 with cultures for fungus, anaerobes, and microbes returning back normal. Hemoglobin S resulted at 20.5%, reflective of a sickle cell trait phenotype from her donor, and the tacrolimus level came back elevated at 19.3 ng/mL, markedly elevated from her last value of 13.6 ng/mL, 3 days prior. The target goal for her tacrolimus therapy was 9–12 ng/mL. A MRI was then conducted and demonstrated restricted diffusion involving the bilateral corona radiata with additional small areas of restricted diffusion involving the right anterior limb of the internal capsule and right occipital white matter as shown in Figure 1. There was no associated T2/FLAIR hyperintensity and no abnormal enhancement. These MRI findings were suggestive of TL due to tacrolimus.

Tacrolimus therapy was discontinued, and the blood level of tacrolimus was trended which decreased to 5.5 ng/mL over the next 4 days. During this time, she returned to her neurological baseline with complete resolution of her aphasia and dysarthria. She was then switched to mycophenolate mofetil for GVHD prophylaxis and was discharged.

## Discussion/Conclusion

Our patient presented with acute onset expressive aphasia after 43 days of tacrolimus prophylactic therapy. Initially, diagnostic hypotheses included acute ischemic stroke, infection due to immunosuppression, GVHD, sickle cell crisis, acute disseminated encephalomyelitis, and medication toxicity. However, the MRI finding of diffuse restricted diffusion with ADC drop out signal involving bilateral corona radiata and areas of white matter in the right cerebral hemisphere was consistent with the diagnosis of tacrolimus induced leukoencephalopathy.

Tacrolimus is a calcineurin inhibitor that blocks T-lymphocyte signal transduction and IL-2 transcription allowing it to be useful in the prevention of GVHD. Mechanisms that may underlie tacrolimus neurotoxicity can be related to the drug being highly lipophilic with the ability to damage the blood-brain barrier and white matter, excess production of endothelin may allow the drug to gain access to astrocytes, and the ability to alter mitochondrial function leading to cell apoptosis.^3^ In addition, tacrolimus is hepatically cleared and any damage or dysfunction of the liver may reduce the plasma clearance of tacrolimus and increase the levels leading to toxicity.

The diagnosis of TL requires a high index of suspicion because the MRI and clinical findings may be similar to other nontoxic causes. As such, when a patient presents with acute neurologic deficits, it is important to obtain a detailed history including exposure to neurotoxic agents. In addition, because one of the earliest complications of immunosuppressive therapy is neurotoxicity due to the narrow therapeutic index it is important to obtain drug levels to determine the cause of the neurologic deficits and hold medications until the level is appropriate. The tacrolimus neurotoxicity that resulted in this patient may have been exacerbated by the patient's liver dysfunction or worsened by the coadministration of methadone, which is also hepatically cleared and can result in TL.

Prior research has indicated that various etiologies exist for causing acute TL including chemotherapeutic and immunosuppressive agents, illicit drug use, and environmental toxins.^4‐8^ Although the exact pathophysiology is still unknown, it is postulated that the neurotoxic properties of many of these causative agents result in white matter dysfunction leading to cytotoxic edema, demyelination, and axonal injury. A recent study by Özütemiz et al found that chemotherapeutic agents such as methotrexate, fludarabine, and 5-fluorouracil, are the most common cause of TL in a study of 101 patients while immunosuppressant therapy induced TL was less likely.^9^ It has also been reported that the prevalence of tacrolimus induced neurotoxicity varies from 5–30% but is more frequent in adults after liver transplantation.^10^ TL is rarer in pediatric patients and the neurotoxicity that results often improves with removal of the drug.^10^

With the improvement in imaging studies and techniques, TL can now be detected much sooner, and the causative agent can be discontinued to prevent possible long term sequelae of this acute brain insult. Brain MRI for TL shows a pattern of bilateral and symmetric white matter areas of hyperintense signal on T2-weighted and fluid-attenuated inversion recovery images, and signs of restricted diffusion are associated in the acute stage which is consistent with the findings in our case.^11^

In conclusion, we describe a post stem cell transplant pediatric patient who presented with stroke-like symptoms due to tacrolimus induced toxic leukoencephalopathy with neuroimaging demonstrating diffuse restricted diffusion involving bilateral corona radiata and areas of white matter in the right cerebral hemisphere. Stroke symptoms improved quickly after discontinuation of the offending agent.
